# Mapping the H^+^ (V)-ATPase interactome: identification of proteins involved in trafficking, folding, assembly and phosphorylation

**DOI:** 10.1038/srep14827

**Published:** 2015-10-07

**Authors:** Maria Merkulova, Teodor G. Păunescu, Anie Azroyan, Vladimir Marshansky, Sylvie Breton, Dennis Brown

**Affiliations:** 1 MGH Center for Systems Biology, Program in Membrane Biology & Division of Nephrology, Richard B. Simches Research Center, Massachusetts General Hospital and Department of Medicine, Harvard Medical School, Boston, MA 02114, USA

## Abstract

V-ATPases (H^+^ ATPases) are multisubunit, ATP-dependent proton pumps that regulate pH homeostasis in virtually all eukaryotes. They are involved in key cell biological processes including vesicle trafficking, endosomal pH sensing, membrane fusion and intracellular signaling. They also have critical systemic roles in renal acid excretion and blood pH balance, male fertility, bone remodeling, synaptic transmission, olfaction and hearing. Furthermore, V-ATPase dysfunction either results in or aggravates various other diseases, but little is known about the complex protein interactions that regulate these varied V-ATPase functions. Therefore, we performed a proteomic analysis to identify V-ATPase associated proteins and construct a V-ATPase interactome. Our analysis using kidney tissue revealed V-ATPase-associated protein clusters involved in protein quality control, complex assembly and intracellular trafficking. ARHGEF7, DMXL1, EZR, NCOA7, OXR1, RPS6KA3, SNX27 and 9 subunits of the chaperonin containing TCP1 complex (CCT) were found to interact with V-ATPase for the first time in this study. Knockdown of two interacting proteins, DMXL1 and WDR7, inhibited V-ATPase-mediated intracellular vesicle acidification in a kidney cell line, providing validation for the utility of our interactome as a screen for functionally important novel V-ATPase-regulating proteins. Our data, therefore, provide new insights and directions for the analysis of V-ATPase cell biology and (patho)physiology.

While the eukaryotic V-ATPase is primarily known as a proton-pumping, rotary nano-motor^1^,^2^ that is involved in many physiological processes^3^,^4^, it also plays an important role in: i) vesicle coat formation^1^ and ii) regulation of signaling and trafficking of various receptors^5^. The V-ATPase was also identified as a sensing and signaling complex that functions in the endocytic pathway^6^,^7^,^8^, and it has a crucial role in SNARE-dependent fission/fusion process and organelle biogenesis along the exocytic pathway^5^,^9^. Within any given cell, the V-ATPase holoenzyme subunit profile differs among organelles and plasma membrane domains^3^,^10^. For example, the 56-kDa B subunit has two isoforms, one ubiquitous (B2) and one that is expressed in specialized proton-secreting cells (B1). In general, the subunit isoforms responsible for activities such as acidification of endosomes and lysosomes are “ubiquitous” and their dysfunction is incompatible with life. V-ATPase activity is essential for post-implantation development^11^, and no disease-causing mutations in these isoforms have been identified. However, loss-of-function mutations of tissue- or cell type-specific^12^ isoforms, can lead to specific, but non-fatal, disease conditions such as osteopetrosis, distal renal tubular acidosis, deafness, osteoporosis^13^, Parkinsonism^14^ and impairment of insulin secretion^15^. Conversely, V-ATPase activity exacerbates metastasis in some cancers and its inhibition is an emerging strategy to block cancer progression^16^. Thus, the possibility of isoform-selective and targeted modulation of enzyme activity is a feasible therapeutic goal.

Various mechanisms by which regulation of V-ATPase activity occurs are known. They include gene and protein expression, subunit assembly and disassembly, recycling of V-ATPase-coated vesicles to and from the plasma membrane, and uncoupling of ATP hydrolysis from proton pumping^2^,^4^,^10^,^17^. However, few of the potential “accessory” proteins involved in these processes have been identified. The aim of the current study was, therefore, to generate a V-ATPase protein-protein interaction roadmap from which regulators of both ubiquitous as well as tissue and cell-type specific proton pumps would emerge. This systematic approach, applied here to the kidney, has allowed us to identify novel interacting proteins that could be involved in V-ATPase quality control, subunit assembly and trafficking, all of which are coordinated to regulate V-ATPase-dependent acidification processes in health and disease states.

## Immunoprecipitation, protein identification and interaction scoring

To identify V-ATPase-associated proteins and construct an interaction network, we co-immunoprecipitated (IP) proteins from mouse kidney lysates using antibodies against the V-ATPase B1 subunit that is part of the multisubunit cytoplasmic V_1_ domain^18^, and identified them by mass-spectrometry. The other major V-ATPase domain is called V_0_, and consists largely of transmembrane subunits. We addressed the question of specificity by performing IPs using beads only (i.e., no B1 antibody), and IPs from B1-deficient mouse kidneys^19^ (Supplementary Table S1). We also used a third type of control (IPpeptide), in which IPs were performed with anti-B1 antibodies that were preincubated with the B1 C-terminal peptide used for antibody generation. We have previously used this method to reveal the interaction between the C-terminal PDZ-domain of the AQP9 water channel and the PDZ protein NHERF1^20^. We showed that the AQP9 peptide containing the PDZ motif is more efficient in immunoprecipitating NHERF1 than the holo-AQP9 protein. This might be due to a better accessibility of the PDZ domain located on the peptide for interaction with NHERF1, versus the holo-AQP9 protein when bound to the antibody. The B1 C-terminus also contains a PDZ binding protein-protein interaction domain^18^, and we used the IPpeptide procedure to reveal proteins that interact through this domain. In addition to serving as a “positive control” for proteins that interact with the B1 C-terminus domain, this procedure can also serve as a negative control for all non-C-terminally bound proteins (ATP6V1B1_ΔCterminus). It is also important to clarify that we cross-linked peptide to antibodies and to beads followed by washing, and there was no excess of B1 C-terminal peptide to antibodies in the IPpeptide experiments. Accordingly IPpeptide experiments were used as additional negative controls for scoring ATP6V1B1_ΔCterminus interactions, but as specific binding experiments for scoring ATP6V1B1_Cterminus interactions.

Immunoprecipitation with anti-ATP6V1B1 antibodies followed by LC-MS/MS analysis was repeated 4 times from 4 different wild-type mice (experiments IP1, IP2, IP3 and IP4); immunoprecipitation with anti-ATP6V1B1 antibodies, pre-incubated with B1 peptide (“B1 peptide pull-down experiment”) and its LC-MS/MS analysis was repeated twice in parallel with IP2 and IP3 experiments from the same wild type mice (experiments IP2peptide and IP3peptide). Beads only negative controls were performed three times, twice in parallel with IP1 and IP4 experiments from the same mouse kidney lysates; and the third time independently from an additional (5th) wild-type mouse (experiments IP1beads, IP4beads and IP5beads). Immunoprecipitation of proteins with anti-ATP6V1B1 antibodies from B1 knockout mouse kidney lysates was repeated 2 times from 2 different B1 knockout mice (experiments IP6KO and IP7KO). Thus, in total 11 experiments were performed; an overview is presented in Supplementary Table S1. Reproducibility of these experiments was approximately 50% in a pairwise comparison of the same type of immunoprecipitations (Supplementary Table S1). All interacting proteins were identified by SEQUEST. A summary of SEQUEST output information, as provided by the Taplin Mass Spectrometry Facility (Harvard Medical School), including peptide sequences, Xcorr scores, number of unique peptides and total spectrum counts can be found in Supplementary Table S2.

**Figure 1:**
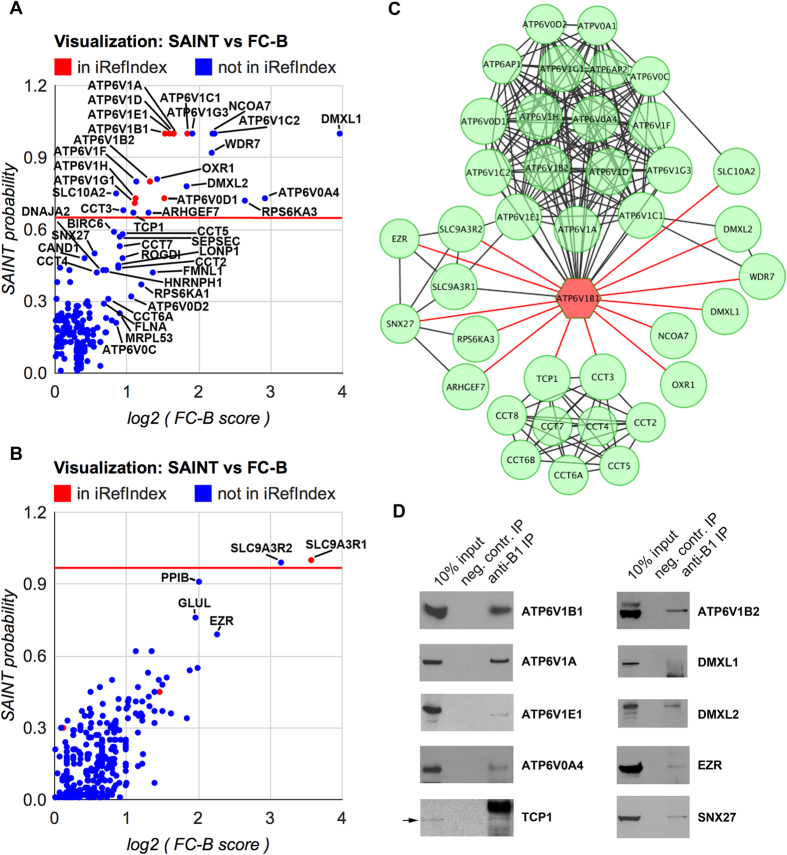
Scoring, construction and validation of the V-ATPase interactome. (**A**) The visualization plot (SAINT vs FC-B) of results of ATP6V1B1_ΔCterminus interaction scoring. Interactions that have been previously reported in iRefIndex database are shown as red circles, unreported interactors are shown as blue circles. Proteins with SAINT ≥ 0.67 are separated by a red line. (**B**) The visualization plot (SAINT vs FC-B) of results of ATP6V1B1_Cterminus interaction scoring. Proteins with SAINT ≥ 0.99 are separated by a red line, other details are as in (**A,C**) V-ATPase interactome constructed from both high-scoring and some lower-scoring interactors identified in this study, as described in the results. Official mouse protein symbols are shown in accordance with nomenclature from the MGI database. Larger circles (nodes) represent proteins with SAINT ≥ 0.67, smaller circles represent proteins with SAINT < 0.67. Previously known physical associations (edges) between proteins are shown in black, novel interactions, found in this study for the first time are shown in red. (**D**) Interactions with representative proteins are confirmed by Western blotting. Proteins were immunoprecipitated by anti-B1 antibodies from whole kidney lysates and then detected by antibodies against the indicated proteins.

Mouse official gene/protein symbols and total spectrum counts were extracted from SEQUEST output information and used to score the interactions by the CRAPome computational tool (http://www.crapome.org/)^21^. First, FC-A, FC-B and SAINT probability scores were calculated for the ATP6V1B1_ΔCterminus interactome. IP1, IP2, IP3 and IP4 experiments served as baits and scored, with the 7 other experiments (IP1beads, IP4beads, IP5beads, IP2peptide, IP3peptide, IP6KO and IP7KO) uploaded as negative controls. We identified 23 high-scoring interactions (SAINT ≥ 0.67, the rational for choosing this threshold is described in Methods), including ATP6V1B1 and 12 other subunits of V-ATPase (Supplementary Table S3, Fig. 1A). Thus, 10 high-scoring proteins that are not core subunits of the V-ATPase complex were identified. All of them were not previously known to interact with B1 subunits of V-ATPase, based on information from iRefIndex (Supplementary Table S3, Fig. 1A). Some of these high-scoring interactions were also confirmed by Western blotting (Fig. 1D) when functional antibodies were available.

Next, FC-A, FC-B and SAINT probability scores were calculated for the ATP6V1B1_Cterminus interactome. In this case, IP2peptide and IP3peptide experiments served as baits and scored, with IP2 and IP3 as negative controls (Supplementary Table S4, Fig. 1B). Here, just two proteins showed a clear separation from the rest of the dataset (SAINT ≥ 0.99 and FC-B ≥ 8.91)—SLC9A3R1 (also known as NHERF1) and SLC9A3R2 (also known as NHERF2). The interaction between V-ATPase and SLC9A3R1 was reported previously in our laboratory using a different technology^18^.

## Network construction and protein clusters

First, 23 high-scoring proteins (including 13 V-ATPase subunits) from the ATP6V1B1_ΔCterminus interactome and 2 high-scoring proteins from the ATP6V1B1_Cterminus interactome were included in the final protein-protein interaction network. Among lower-scoring interactors there were 5 more V-ATPase subunits. Because the V-ATPase is a well-characterized protein complex, these were added to the final network. 2 subunits of the CCT (chaperonin containing TCP1) complex were among high-scoring interactors. We included 7 lower scoring subunits of the CCT complex into the network based on the fact that CCT is also a well characterized stable complex. SNX27 and EZR were below the threshold for inclusion based on their SAINT score alone, but we had detected their interaction with the V-ATPase by Western blotting (Fig. 1D) and we, therefore, included them in the V-ATPase interactome. In this way, a total of 18 V-ATPase subunits, 9 CCT subunits, and 12 other V-ATPase interacting proteins are included as 39 nodes in Fig. 1C, drawn in Cytoscape^22^.

V-ATPase and CCT complexes are visualized as sub-networks, where each individual subunit is connected to all other subunits of the corresponding complex. Other connections (shown as edges) are based on this study and previously published high-throughput and low-throughput experiments as identified by QIAGEN’s Ingenuity® Pathway Analysis (IPA®, QIAGEN, Redwood City, CA, www.qiagen.com/ingenuity). IPA revealed that apart from V-ATPase and CCT complexes, 6 proteins (ARHGEF7, RPSKA3, SNX27, EZR, SLC9A3R1 and SLC9A3R2) are interconnected with each other, forming a distinct cluster. The role of these proteins in trafficking is very well established and may not be specific to V-ATPase. Five members of the other group (SLC10A2, DMXL1, DMXL2, WDR7, NCOA7 and OXR1) (with the exception of SLC10A2) are not well characterized functionally and we propose that they are novel V-ATPase specific accessory proteins, as also discussed below. The final list of proteins, included in our interactome and classified by their proposed functional role with regard to the V-ATPase is shown in Table 1.

To compare our results with previously published data we found first neighbors for all known subunits of the V-ATPase (totally 25 proteins) with the IPA tool (Supplementary Fig. S1). A total of 176 proteins were previously reported to interact with various subunits of V-ATPase, 64 of which had been reported to interact with the B1 subunit of V-ATPase. Some of these proposed interactions are from high-throughput experiments, while the others are from low-throughput experiments and are characterized in more detail. It is clear that even this long list of previously reported V-ATPase-interacting proteins is far from complete, because even some of the subunits of V-ATPase (ATP6V0B, ATP6V0E2 and ATP6V1C2) are not connected to the V-ATPase protein-protein interaction network (Supplementary Fig. S1). One of the reasons is that IPA restricts data to just three species, human, mouse and rat, while much V-ATPase research has been carried out on non-mammalian species, particularly yeast. On the other hand, many of these 176 published V-ATPase protein-protein interactions might be non-specific and/or non-functional, because proper controls were not always performed, especially in the high-throughput experiments. Among our 10 high-scoring (SAINT ≥ 0.67) interactors, 5 (DMXL2, WDR7, SLC10A2, SLC9A3R1 and SLC9A3R2) have been reported previously to interact with different subunits of V-ATPase, and only SLCA3R1 with the B1 subunit (Fig. 1C, Supplementary Fig. S1, circled in red). By considering lower interaction scores (SAINT < 0.67) we identified 37 more of the previously reported V-ATPase interacting proteins (Supplementary Fig. S1, circled in green). Among them there are previously well-characterized interactors including ACTB, ALDOA, ALDOB, AP2B1, AP2M1, ARF6, PFKL, PFKM and PFKP^1^,^2^. The major reason for the low scoring of these proteins in our study is their high degree of non-specific binding in the negative controls (Supplementary Table S3). The complete list of previously published interactions has been compared to our present results and is presented in Supplementary Table S5.

### Transporters

As mentioned above, 18 out of 25 known subunits of the V-ATPase were identified in this analysis (Table 1). 5 of these (ATP6V0D2, ATP6V0C, ATP6AP1, ATP6AP2, ATP6V0A1) were scored with low probability (SAINT < 0.67). All are from the transmembrane V0 sector of the V-ATPase and based on current structural models do not interact with the V-ATPase B1 subunit directly. In addition, there may be isoform preference of subunit binding. For example, the B1 subunit preferentially binds the ATP6V0A4 subunit isoform but not ATP6V0A1^23^. Of the remaining 7 of the 25 known subunits of V-ATPase that were not detected at all in our study, two are testis-specific ATP6V1E2 and brain-specific ATP6V1G2, and are not expressed in kidney, while ATP6V0A2 and ATP6V0A3 (known as TCIRG1 in mice) ‘a’ subunit isoforms are expressed at considerably lower levels than ATP6V0A4 isoform and preferentially bind B2 (ATP6V1B2) and not B1 (ATP6V1B1) subunit isoform^23^. The remaining three undetected subunits are transmembrane proteins ATP6V0B, ATP6V0E1 and ATP6V0E2 and do not interact with the B1-subunit of V-ATPase directly. The ubiquitously expressed (B2, C1 and G1) and kidney-specific (B1, C2, G3 and a4) isoforms of the V-ATPase B-, C-, G- and a-subunits were all identified as high-scoring interactors, indicating that they are possibly present in some “hybrid” V-ATPase complexes (Supplementary Table S3 and Fig. 1A). Unexpectedly, the kidney-specific d2 subunit was identified as a low-scoring interactor, while the ubiquitous d1 subunit was high-scoring (Supplementary Table S3 and Fig. 1A). Thus, based on these data, the B1 subunit of V-ATPase preferably associates with d1 and not with the d2 subunit. Interestingly, a d2 subunit knockout mouse did not demonstrate renal function abnormalities^24^.

Surprisingly, the only kidney transporter to interact with the V-ATPase with high confidence was SLC10A2, also known as ASBT, an apical sodium-dependent bile acid transporter. While this association was reported in the literature more than 10 years ago^25^, its functional significance is still unclear. Since V-ATPase is known to “energize” other transporters in the kidney^26^, it is possible that bile acid reabsorption from urine may be energized by the V-ATPase-driven proton gradient.

### Chaperonin containing TCP1 complex (CCT)

We also reproducibly detected all known subunits of the chaperonin containing TCP1 complex (TCP1, CCT2-8)^27^ although with both higher and lower than threshold SAINT scores. We propose here that this complex is involved in folding and quality control of V-ATPase molecules and contributes to overall V-ATPase expression levels and activity. Several proteins involved in folding and assembly of V-ATPase subunits have been identified in yeast^2^,^17^ but in mammalian cells, only a VMA21 homologue has been identified so far, its deficiency causing autophagic vacuolar myopathy in humans^28^.

### Trafficking

Among proteins involved in V-ATPase trafficking are the scaffolders SLC9A3R1, SLC9A3R2, a member of the ERM protein family EZR, the sorting nexin SNX27, RAC1 GDP/GTP exchange factor ARHGEF7 and a member of the S6 serine/threonine protein kinase subfamily RPS6KA3. All these proteins are well characterized but except for SLC9A3R1 none of them was previously connected to the regulation of V-ATPase trafficking. Three of these proteins (SLC9A3R1, SLC9A3R2 and EZR) are proposed to link V-ATPase to the cytoskeleton^29^,^30^. It has been reported that the B1, B2 and C subunits of V-ATPase directly bind to actin^29^,^31^,^32^ and that the actin remodeling protein gelsolin is involved in regulation of V-ATPase recycling^30^. Based on our data, linkage of V-ATPase to the actin cytoskeleton could occur through SLC9A3R1/SLC9A3R2 homo- and heterodimers, via EZR. While we routinely detected actin and tubulin in our co-IPs, they were also present in negative controls in comparable amounts, making it difficult to assign specificity to their association with the V-ATPase (Supplementary Table S3).

Three proteins mentioned above—SNX27, SLC9A3R1 and SLC9A3R2 – contain PDZ protein-protein interaction domains. Through a PDZ domain, SNX27 binds to the C-terminus of various transmembrane proteins and promotes their recycling from endosomes to the plasma membrane^33^. It is likely that the association between V-ATPase and SNX27 also occurs through direct binding of SNX27 to the C-terminal PDZ-binding motif of the V-ATPase B1 subunit. SLC9A3R1 and SLC9A3R2 each contain two PDZ domains, and SLC9A3R1 was previously shown to bind directly to the DTAL PDZ-binding motif of V-ATPase B1^18^. The relative contribution of these PDZ proteins to V-ATPase regulation remains to be determined.

Finally, our network highlights two upstream regulators of V-ATPase trafficking as well as its interactions with the cytoskeleton. These include RAC1 GDP/GTP exchange factor ARHGEF7 and a serine/threonine protein kinase RPS6KA3. We previously reported that RHOA and its effector ROCKII are implicated in V-ATPase membrane redistribution^4^, and our current data now implicate ARHGEF7 and RAC1 in this process. Phosphorylation is one of the mechanisms of regulation of V-ATPase trafficking: while various V-ATPase subunits are phosphorylated^34^,^35^, only two of the potential kinases involved have been identified^36^. Our new data suggest that RPS6KA3 may also phosphorylate V-ATPase subunits.

### Novel V-ATPase specific accessory proteins

**Figure 2:**
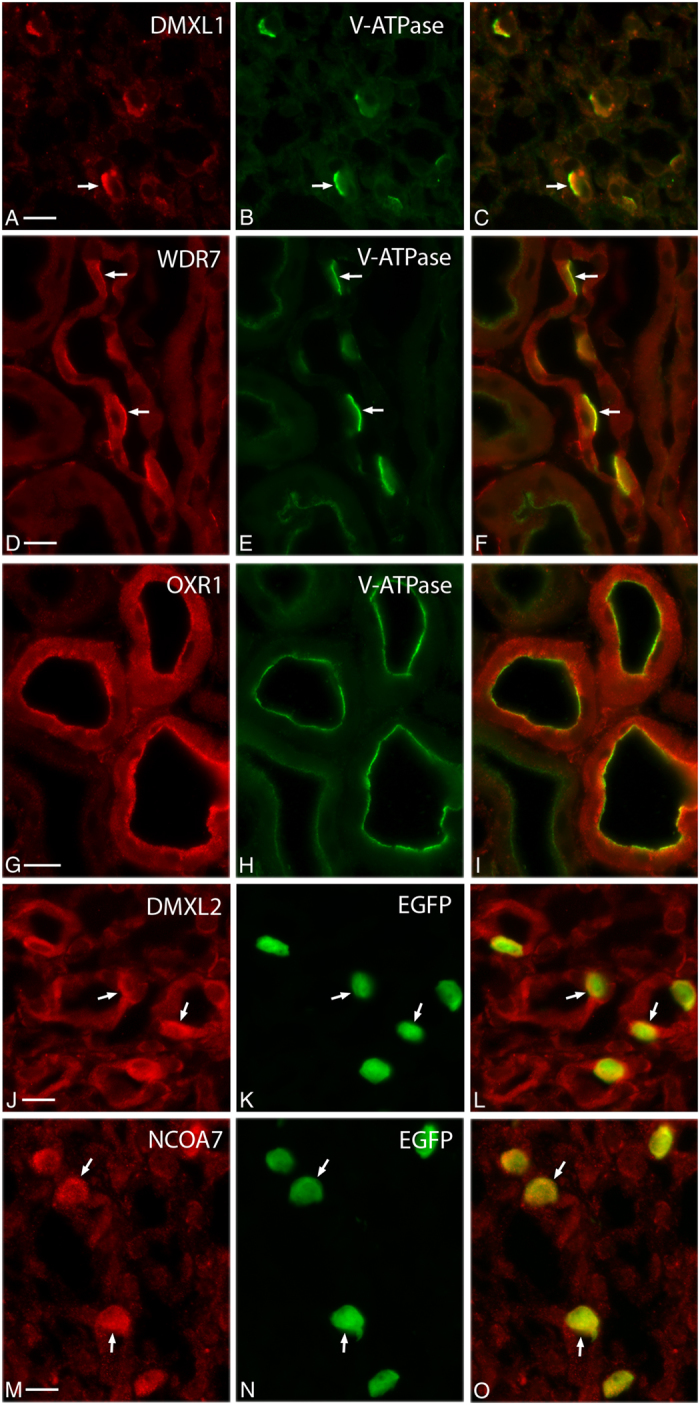
Localization of DMXL1, DMXL2, NCOA7, OXR1 and WDR7 in proton secreting cells of mouse kidney by immunocytochemistry. DMXL1 ((**A)**, red) co-localizes with the A-subunit of V-ATPase ((**B**), green) in intercalated cells (ICs, indicated with arrows) of wild type mouse inner medullary collecting duct. WDR7 ((**D**), red) also co-localizes with the same V-ATPase subunit (**E**), green) in ICs of wild type mouse cortical collecting duct. OXR1 (**G**), red) and V-ATPase ((**H**), green) co-localize in the apical pole of distal convoluted tubule cells of wild type mice. DMXL2 (**J**), red) and NCOA7 (**M**), red) are expressed predominantly in ICs of B1-EGFP transgenic mouse inner medullary collecting duct (ICs are green in panels (**K**,**N**)). Note, that in the B1-EGFP transgenic mouse EGFP is not fused to B1 subunit, rather EGFP expression is driven by B1 promoter. Thus, while EGFP is expressed specifically in ICs, its localization in these cells is purely cytosolic and does not follow V-ATPase sub-cellular localization pattern. Merged images (**C,F,I,L,O**) are shown in the right column. Scale bar = 10 μm.

Finally, we found that five insufficiently characterized proteins (DMXL1, DMXL2, NCOA7, OXR1 and WDR7) are associated with the V-ATPase (Fig. 1C, right). These proteins have the highest SAINT scores among all interacting proteins. They were scored as high as several subunits of the V-ATPase themselves. This suggests that they may be highly specific “accessory” proteins of the V-ATPase. We confirmed that all five proteins co-localize with the V-ATPase in kidney proton secreting cells, such as collecting duct intercalated cells, which are involved in urinary acidification^3^,^4^. Intercalated cells in wild type mice were identified by anti-V-ATPase immunostaining, which overlaps with DMXL1 (Fig. 2A–C) and WDR7 staining (Fig. 2D–F) in the apical membrane and subapical domain of these cells. OXR1 co-localizes with the V-ATPase in a similar pattern in intercalated cells, as well as in cells of the distal convoluted tubule (Fig. 2G–I). In mice in which EGFP expression is driven by the promoter of the V-ATPase B1 subunit^37^, intercalated cells were identified by EGFP expression (Fig. 2K,N). In these cells, EGFP is cytosolic, because it is not directly linked to the B1 V-ATPase subunit. As we described previously, the subcellular V-ATPase localization in these cells is similar to the localization pattern seen in intercalated cells of wild type mice^37^,^38^. Accordingly, DMXL2 (Fig. 2J–L) and NCOA7 (Fig. 2M–O) are co-expressed and also co-localize, at least partially, with V-ATPase in these cells.

**Figure 3:**
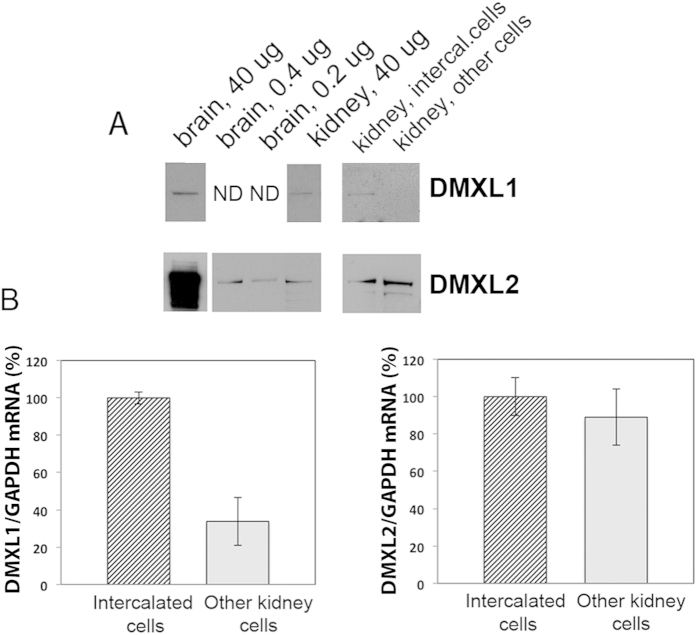
DMXL1 expression is upregulated in kidney intercalated cells, and DMXL2 is highly expressed in brain. (**A**) Western blotting showing similar DMXL1 protein levels in whole brain in comparison with whole kidney; in kidney DMXL1 protein was detected in intercalated cells, but not in the other cell types. In contrast, DMXL2 protein is very highly expressed in whole brain and at significantly lower (approximately 100-fold difference) levels in whole kidney, where no significant difference of expression was detected between intercalated and other cell types. ND—not determined. (**B**) Real-time qRT-PCR analysis showing approximately 4 fold increase in DMXL1 mRNA expression level in intercalated cells in comparison with other cell types, but no significant difference in DMXL2 mRNA expression levels between these cell types.

The expression of DMXL1 and DMXL2 in intercalated cells was further examined by Western blotting and RT-PCR. EGFP(+) intercalated cells were isolated by fluorescence-activated cell sorting from B1-EGFP mice. EGFP(−) cells were also collected and represent a highly heterogeneous population of all other kidney cell types. DMXL1 and DMXL2 protein were both detected in whole kidney and brain (used as a positive control)^39^ (Fig. 3A). There was no significant difference in expression of DMXL1 protein in whole kidney in comparison with whole brain, while DMXL2 protein was expressed in kidneys at much lower levels (~100 fold difference), than in brain (Fig. 3A). In contrast, there was a ~4-fold increase in DMXL1 mRNA expression in GFP(+) cells compared to GFP(−) cells (Fig. 3B) and, DMXL1 protein was detectable only in GFP(+) cells (Fig. 3A). There was no significant difference in expression of DMXL2 mRNA or protein in GFP(+) compared to GFP(−) cells (Fig. 3A,B). These data confirm the higher specificity of DMXL1 for intercalated cells versus principal cells that we found by immunofluorescence, as well as the presence of DMXL2 in principal cells in addition to intercalated cells (Fig. 2). Immunocytochemistry also revealed a somewhat greater DMXL2 staining in GFP-positive intercalated cells than neighboring cells, which were also positively stained (Fig. 2). In summary, we found that DMXL1 expression is high in specialized proton-secreting cells in the kidney. DMXL2 is highly expressed in brain, but is also detectable in the kidney.

**Figure 4:**
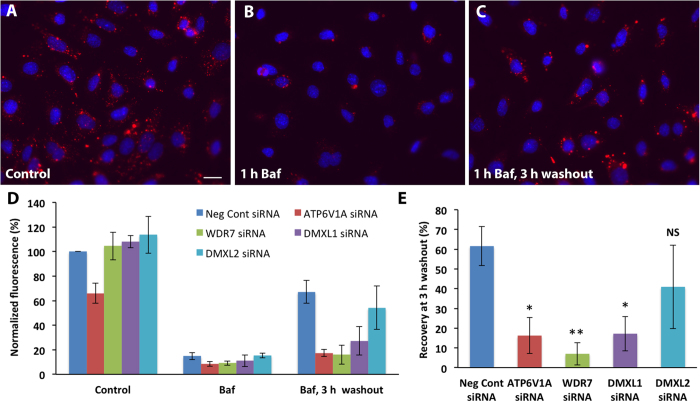
WDR7 and DMXL1, but not DMXL2 siRNA knockdown significantly decreases V-ATPase-dependent re-acidification of intracellular vesicles during recovery from bafilomycin A1 (Baf) treatment in M-1 mouse cortical collecting duct cells. Under steady-state (control) conditions M-1 cells are readily labeled with lysosomotropic red-fluorescent dye LysoTracker Red DND-99 as visualized by fluorescence microscopy (**A**) and quantified by fluorimetry ((**D)**, Control). ATP6V1A siRNA, targeting the catalytic A subunit of V-ATPase, was used as a positive control and it is the only siRNA which decreases vesicle acidification of intracellular vesicles under steady-state conditions, although not as potently as Baf ((**D**), Control and Baf). After pretreatment with 100 nM Baf for 1 hour the number of acidic vesicles (**B**) and total fluorescence intensity ((**D**), Baf) are reduced dramatically in all cases, and both slowly recover to various degrees after Baf is washed out ((**C,D**), Baf, 3 h washout). (**E**) The inhibition of re-acidification is statistically significant for ATP6V1A, WDR7 and DMXL1, but not for DMXL2 siRNA treated cells. The recovery was calculated as a ratio of each Baf-subtracted value at 3 hours after Baf washout to the corresponding Baf-subtracted untreated control values and shown as a percentage. All data are presented as mean values and error bars indicate the standard deviation. *P < 0.01, **P < 0.001 relative to negative control siRNA. NS – non significant. Scale bar = 10 μm.

To determine if DMXL1, DMXL2 and WDR7 play a role in V-ATPase function, we used an siRNA knockdown approach in M-1 mouse cortical collecting duct cells, which express these proteins. siRNA against the ATP6V1A catalytic subunit of the V-ATPase was used as a positive control. Efficient siRNA knockdown was confirmed by quantitative real-time PCR and in all cases the level of knockdown was similar, about 80% at the mRNA level. After siRNA treatment, M-1 cells were labeled with the lysosomotropic red-fluorescent dye LysoTracker® Red DND-99 as a readout of V-ATPase-driven intravesicular acidification. This dye is freely permeant across cell membranes at neutral pH, but becomes protonated and trapped in acidic compartments. As shown in Fig. 4A, DND-99 readily labeled acidic vesicles in M-1 cells under control steady-state conditions. Cells were transfected with siRNAs against DMXL1, DMXL2, WDR7, ATP6V1A or negative control siRNAs for 72 hours, labeled with DND-99, lysed and the released total fluorescence intensity was then measured using a fluorometer, as described in methods. Under baseline conditions, there was no significant difference in the total fluorescence intensity and therefore intravesicular acidification between DMXL1, DMXL2 or WDR7 siRNA transfected cells and the negative control siRNA treated cells (Fig. 4D, Control). ATP6V1A siRNA treatment decreased acidification by 34% on average, suggesting either that the amount of ATP6V1A subunit remaining after knockdown is sufficient to sustain vesicle acidification under steady-state conditions, or that there may be some compensatory feedback mechanisms. We, therefore, used a pH recovery approach to evaluate the role of these proteins in V-ATPase-dependent vesicle acidification after exposure to the highly specific V-ATPase inhibitor bafilomycin. A one-hour treatment with 100 nM bafilomycin inhibited DND-99 accumulation in all cases (Fig. 4B,D, 1 h Baf), indicating significant impairment of acidification. After bafilomycin washout for 3 h, dye accumulation was clearly detectable in negative control siRNA treated cells, indicating re-acidification. However, it was suppressed to various degrees in DMXL1, DMXL2, WDR7 or ATP6V1A siRNA treated cells (Fig. 4C,D, 1 h Baf, 3 h washout). The decrease in V-ATPase-dependent recovery of acidification was statistically significant for DMXL1, WDR7 and ATP6V1A siRNA treated cells when compared to negative controls (Fig. 4E). DMXL2 siRNA treatment caused a decreasing trend but it was variable and not statistically significant (Fig. 4E). It is possible that DMXL1 compensates for the knockdown of DMXL2, which is more highly expressed in the brain than the kidney (Fig. 3A). These data suggest that DMXL1, DMXL2 and WDR7 play an essential role in situations that require robust and sustained upregulation of V-ATPase activity. However, it has been reported previously, that in HaCaT keratinocytes and MCF-7 breast cancer cells, WDR7 siRNA knockdown reduces DND-99 staining of acidic vesicles even under steady-state conditions^40^; thus the degree of WDR7 requirement for V-ATPase function could be also cell-specific.

DMXL2, also known as rabconnectin 3 A, was first identified by co-immunoprecipitation with RAB3 GEF and GAP from brain synaptic vesicles, and its role in RAB3-mediated exocytosis has been shown^39^. The same group found that WDR7 associates with rabconnectin 3 A and directly interacts with RAB3 GEF but not GAP^39^. Studies in Drosophila, zebrafish and mammalian cell lines showed that rabconnectins are required for V-ATPase-driven acidification^4^. Based on its homology with the ubiquitous DMXL2, we propose that DMXL1 plays a similar role in regulating V-ATPase exocytosis but functions in specific proton-secreting cell types, such as kidney intercalated cells. NCOA7 was originally discovered as an estrogen nuclear receptor coactivator but it also interacts with autophagosomal proteins^41^ and with RAB3GAP1^42^. OXR1 was originally discovered due to its ability to reduce the DNA damaging effects of reactive oxygen species in bacteria, and subsequently it was shown to be induced by stress and localized to mitochondria in yeast and human cells^43^,^44^. In spite of a striking difference in subcellular localization, regulation and function, NCOA7 and OXR1 are homologues. Here we found a new common feature between these two proteins: they both interact with V-ATPase. Based on these data, we suggest that DMXL1, DMXL2, WDR7, NCOA7 and OXR1 may link V-ATPase to the RAB3 subfamily of small GTPases, which are involved in regulating exocytosis and V-ATPase activity^45^,^46^.

DMXL1 and DMXL2 are partially homologous to the yeast protein RAV1 (regulator of V-ATPase in vacuolar membrane protein 1)^17^. RAV1 is the central component of the RAVE complex, which is required for assembly of the V_1_ and V_0_ sectors in yeast^17^. Whether DMXL1 and DMXL2 promote assembly of the V-ATPase in mammalian cells will require further investigation.

DMXL1, DMXL2 and WDR7 but not NCOA7 nor OXR1 contain at least two WD40 protein-protein interaction domains, often present in scaffolding proteins. It is highly plausible that these proteins directly interact with certain subunits of V-ATPase through WD40 domains and either control assembly/disassembly of V-ATPase as shown for the yeast homologue RAV1, and/or together with NCOA7 and OXR1 regulate V-ATPase exocytosis through scaffolding to RAB3 as discussed above.

In conclusion, *de novo* construction of a V-ATPase protein-protein interaction network has provided many new clues regarding the molecular and cell biology of V-ATPases and their regulation. We revealed several proteins and one protein complex with previously unsuspected V-ATPase-related roles, such as facilitated folding of V-ATPase molecules, phosphorylation and regulation of V-ATPase intracellular trafficking. The mechanism by which V-ATPase-coated vesicles traffic and recycle in proton secreting cells is poorly understood, but RAB3 and associated proteins may be important players. A role for SNARE proteins in V-ATPase exocytosis has also been proposed^9^. While we were able to identify some SNARE complex proteins (such as VAMP8) in our experiments, they had low interaction scores, possibly because their association with V-ATPase is weak or transient. At the molecular level, two protein-protein interaction domains—PDZ and WD40—are highly enriched in V-ATPase network proteins, and it is plausible that these domains are involved in scaffolding V-ATPase to its upstream regulators and downstream effectors. The V-ATPase is increasingly being identified as a drug target in the treatment of various disease conditions, including cancer^16^. The current hope in the V-ATPase field is to find drugs specific for certain cellular pools of V-ATPase molecules, through targeting the specific, non-ubiquitous subunit isoforms. In addition to providing important new avenues for the exploration and dissection of V-ATPase function, the novel protein-protein interactions identified by our study could ultimately serve as potential alternative drug targets for the therapeutic regulation of proton secretion.

## Methods

### Mice

C57BL/6 J (wild-type) mice (Jackson Laboratory, Bar Harbor, ME), B1 knockout^19^ and B1 promoter EGFP-transgenic mice^37^ were housed under standard conditions and maintained on a standard diet. All animal studies were approved by the Massachusetts General Hospital Subcommittee on Research Animal Care, in accordance with the NIH, Department of Agriculture, and Association for the Assessment and Accreditation of Laboratory Animal Care requirements.

### Antibodies

Rabbit and chicken polyclonal antibodies against ATP6V1B1 and ATP6V1B2^47^,^48^; rabbit polyclonal anti-ATP6V1A and anti-ATP6V0A4 antibodies^6^ and chicken polyclonal anti-ATP6V1E1 antibodies^18^ were produced, affinity purified and characterized previously in our laboratory. Chicken polyclonal anti-ATP6V1A antibody was produced and purified using the same peptide as described previously for rabbit polyclonal anti-ATP6V1A antibody^6^. Commercial, affinity purified antibodies used in the study were: rabbit polyclonal anti-DMXL1 (Abnova, Taipei City, Taiwan, PAB23000 and Novus Biologicals, Littleton, CO, NBP1-90998, 1:50), rabbit polyclonal anti-DMXL2 (Novus Biologicals, NBP1-93618, 1:50), rabbit polyclonal anti-NCOA7 (Novus Biologicals, NBP1-85200, 1:100), rabbit polyclonal anti-OXR1 (Thermo Scientific, Waltham, MA, PA5-31746, 1:50), rabbit polyclonal anti-WDR7 (Santa Cruz Biotechnology, Dallas, TX, sc-85210, 1:50), rabbit polyclonal anti-EZR (Cell Signaling Technology, Danvers, MA, 3145, 1:2500), goat polyclonal anti-SNX27 antibody (Santa Cruz Biotechnology, sc-162250, 1:500) and goat polyclonal anti-TCP1 (Santa Cruz Biotechnology, sc-13869, 1:50).

### Immunoprecipitation, LC-MS/MS and protein identification

Wild type C57BL/6 J and B1 knockout^19^ adult male mice were anesthetized with sodium pentobarbital (Nembutal, Abbott Laboratories, Abbott Park, IL, 50 mg/kg body weight, intraperitoneally) and phosphate-buffered saline (PBS) was perfused at a constant rate of 17 ml/min through the cardiac left ventricle to clear the organs of blood. Kidneys were dissected and immediately homogenized in ice-cold lysis buffer (20 mM Hepes-NaOH, pH 7.5; 150 mM NaCl; 1% NP-40), containing Complete Protease inhibitor cocktail (Roche Applied Science, Indianapolis, IN). Kidney lysate was clarified by centrifugation followed by filtration through a 0.2 μm Acrodisc Syringe Filter (PALL Life Sciences, Port Washington, NY, PN4454) and either used immediately for immunoprecipitation or aliquoted and kept frozen at −80 °C. Anti-ATP6V1B1 antibodies were crosslinked to Protein A/G Plus agarose resin using disuccinimidyl suberate and immunoprecipitation was performed following the instructions of Pierce Crosslink Immunoprecipitation Kit (Thermo Scientific, 26147). For each immunoprecipitation experiment, 50 μg of anti-ATP6V1B1 antibody was used. For some experiments prior to crosslinking, 50 μg of anti-ATP6V1B1 antibody was pre-incubated with 100 μg of B1 peptide at approximately 200-fold molar excess of peptide to antibody, and then both peptide and anti-ATP6V1B1 antibodies were crosslinked to Protein A/G Plus agarose resin and the excess antibodies and peptide was then washed off. Thus, there is no excess of peptide to antibodies in the following procedures. Of note, the same peptide was used for the production and purification of anti-ATP6V1B1 antibodies^47^. The immunoprecipitated proteins were separated by SDS-PAGE. Three gel slices per experiment with proteins at different molecular weight ranges were excised and submitted to the Taplin Mass Spectrometry Facility. Mass-spectrometry and protein identification were performed at the facility, as described next. Excised gel bands were cut into approximately 1 mm^3^ pieces and subjected to a modified in-gel trypsin digestion procedure^49^. Gel pieces were washed and dehydrated with acetonitrile for 10 min. followed by removal of acetonitrile. Pieces were then completely dried in a speed-vac. Rehydration of the gel pieces was done with 50 mM ammonium bicarbonate solution containing 12.5 ng/μl modified sequencing-grade trypsin (Promega, Madison, WI) at 4 °C. After 45 min., the excess trypsin solution was removed and replaced with 50 mM ammonium bicarbonate solution to just cover the gel pieces. Samples were then placed in a 37 °C room overnight. Peptides were later extracted by removing the ammonium bicarbonate solution, followed by one wash with a solution containing 50% acetonitrile and 1% formic acid. The extracts were then dried in a speed-vac (~1 hr). The samples were then stored at 4 °C until analysis. On the day of analysis the samples were reconstituted in 5–10 μl of HPLC solvent A (2.5% acetonitrile, 0.1% formic acid). A nano-scale reverse-phase HPLC capillary column was created by packing 5 μm C18 spherical silica beads into a fused silica capillary (125 μm inner diameter × ~20 cm length) with a flame-drawn tip^50^. After equilibrating the column each sample was loaded via a Famos auto sampler (LC Packings, San Francisco, CA) onto the column. A gradient was formed and peptides were eluted with increasing concentrations of solvent B (97.5% acetonitrile, 0.1% formic acid). As peptides eluted they were subjected to electrospray ionization and then entered into an LTQ Velos ion-trap mass spectrometer (ThermoFisher). Peptides were detected, isolated, and fragmented to produce a tandem mass spectrum of specific fragment ions for each peptide. Totally, 33 RAW MS files (3 per each immunoprecipitation and negative control) were obtained and deposited into the MassIVE repository (the Center for Computational Mass Spectrometry at University of California, San Diego (UCSD), http://massive.ucsd.edu) under MassIVE ID MSV000079061. Peptide sequences (and hence protein identity) were determined by matching IPI and UNIPROT protein databases with the acquired fragmentation pattern by the software program, SEQUEST (ThermoFisher)^51^. Spectral matches were manually examined and carry over proteins and common contaminants (such as keratins and trypsin) were removed. The resultant lists of proteins and peptides identified by SEQUEST and provided by the facility are summarized in Supplementary Table S2.

### Interaction scoring

To distinguish between true (specific) and false (non-specific) V-ATPase interactions we scored them using recently developed computational tools freely accessible online at http://www.crapome.org/ (the CRAPome)^21^. This software uses spectrum counts from LC-MS/MS immunoprecipitation experiments and matching negative controls to perform label-free quantification of protein-protein interactions. It first normalizes spectrum counts to the length of the proteins and to the total number of spectra in the purification, and then calculates three scores: two empirical scores (FC-A and FC-B fold changes) and one theoretical score (SAINT probability) based on the entire dataset across all experiments and controls^52^. FC-A score is defined as the ratio of the normalized spectrum count of the co-immunoprecipitated protein, to the average normalized spectrum count of that protein across the negative controls. FC-B score is more conservative than FC-A score and is computed as the ratio of the normalized spectrum count of the co-immunoprecipitated protein, to the average of three highest normalized spectrum counts of that protein across all negative controls. This score is useful when some proteins spike to high abundance in some but not all negative controls and therefore can be underestimated during averaging. Finally, SAINT converts normalized spectrum counts into the probability of a true interaction between the two proteins by modeling of spectrum count distributions for true and false interactions, specific to each protein-protein interaction pair. In addition, the CRAPome maps the interactions to those previously discovered and deposited in the iRefIndex^53^, which provides feedback on the scoring performance, as these known interactions should be given high scores.

To score ATP6V1B1_ΔCterminus interactions, datasets of IP1, IP2, IP3 and IP4 immunoprecipitations and 7 negative control datasets (IP1beads, IP4beads, IP5beads, IP2peptide, IP3peptide, IP6KO and IP7KO) were uploaded to the CRAPome (workflow 3). Only our own negative controls were used for analysis, because there were no matching (to mouse kidney) controls in the CRAPome database. The following analysis options were chosen: 1) for FC-A: Choice of Controls = user controls, Background Estimation = default, Combining Replicates = average; 2) for FC-B: Choice of Controls = user controls, Background Estimation = stringent, Combining Replicates = geometric; 3) for SAINT: Choice of Controls = user controls, Combining Replicates = average, Number of Virtual Controls = 10, n-burn = 2000, n-iter = 4000, LowMode = 0, MinFold = 1, and Normalize = 1. FC-A, FC-B and SAINT scores were computed independently for each of 4 replicates and then averaged. Averages are reported as final scores and are shown on the right side of Supplementary Table S3. The visualization plot (SAINT vs FC-B) is shown in Fig. 1A.

Selection of the threshold for SAINT score was based on the fact that other V-ATPase subunits are true positive interactors, since they all form a well-characterized protein complex. Some of them were indeed highly scored (SAINT = 1), but others had lower scores. Since most V-ATPase subunits had SAINT ≥ 0.71, we assumed then that all proteins with SAINT ≥ 0.71 are true positive interactors. In addition, there was a clear separation of proteins with SAINT ≥ 0.67 from the rest of the dataset (next closest score was 0.59 for BIRC6). Based on these observations, all proteins with SAINT ≥ 0.67 were included in the final V-ATPase interactome. Of note, since some of the subunits of V-ATPase had very low scores (even 0) in spite of the fact that they are true interactors; some other true but low-scoring (probably weak or transient) interactions could be missed even with this relatively low threshold.

Scoring of ATP6V1B1_Cterminus interactions identified in experiments IP2peptide and IP3peptide was performed in a similar way. We tried two different sets of negative controls to score these interactions. First, as negative controls we used only corresponding IP experiments with anti-ATP6V1B1 antibodies, not blocked with B1 peptide, which were obtained from the same mouse kidney lysates (experiments IP2 and IP3). Thus, in this case we had just two replicates of ATP6V1B1_Cterminus interactions and two replicates of negative controls from two different animals. In this case there was a clear separation from the rest of the dataset of just two proteins: SLC9A3R1 (SAINT = 1, FC-B = 11.87) and SLC9A3R2 (SAINT = 0.99, FC-B = 8.91). The next closest protein hit was PPIB (SAINT = 0.91, FC-B = 4.02). Complete results are shown on the right side of Supplementary Table S4 and visualized in Fig. 1B. We also scored IP2peptide and IP3peptide datasets with 9 negative controls. All other experiments (IP1beads, IP4beads, IP5beads, IP1, IP2, IP3, IP4, IP6KO and IP7KO) were uploaded as negative controls. This resulted in spreading of the SAINT and FC-B scores, and many more proteins had high SAINT probability scores. Nevertheless, there was still a clear separation of SLC9A3R1 and SLC9A3R2 based on their high FC-B score (data not shown). Thus, only these two proteins are included in the final V-ATPase protein-protein interaction network.

### RNA isolation and quantitative RT-PCR

Isolation of intercalated (EGFP-positive, EGFP(+)) and non-intercalated (EGFP-negative, EGFP(−)) cells was performed as described previously^54^. RNA extraction was performed immediately with RNeasy mini kit (Qiagen, Valencia, CA) according to the manufacturer’s protocol. Reverse transcription (RT) and PCR reagents were purchased from Applied Biosystems (Foster City, CA). 1 μg or less RNA was reverse transcribed into cDNA for each reaction of 50 μl consisting of 1 × buffer II, 5 mM MgCl_2_, 1.0 mM each dNTP, 1 U/μl RNase inhibitor, 2.5 μM random hexamers, and 2.5 U/μl MuLV reverse transcriptase. Quantitative real-time PCR was performed on reverse transcribed products. The sequences of the PCR primer sets, purchased from Invitrogen (Grand Island, NY), are the following: mouse DMXL1 F 5′-GCATGTTTGAAGATTCTCACCGT-3′; mouse DMXL1 R 5′-CTTTCTCAAGCCAGTGGTAGAG-3′; mouse DMXL2 F 5′- CTGGGGACAACTGCTATTCGG-3′; mouse DMXL2 R 5′-CACCAGGGATGATCTGCACAC-3′; mouse WDR7 F 5′-CCAACAGCAACGAACCTCTTA-3′; mouse WDR7 R 5′-GGCAGGCACAATAATGATGCTT-3′. Real Time PCR (7300 Real Time PCR system, Applied Biosystems) was performed using the Power SYBR Green PCR master mix (Applied Biosystems) according to the manufacturer’s instructions. Amplification products were detected using Standard-curve relative quantifications. Relative values of each sample were normalized to GAPDH values. Samples were analyzed in triplicate for each experiment.

### siRNA transfections and quantitative fluorescence-based vesicle acidification assay

Stealth pre-designed siRNAs (set of three), each targeting three different regions of mouse DMXL1, DMXL2, WDR7 or ATP6V1A mRNAs and one nontargeting Stealth siRNA (negative control) were purchased from Invitrogen. The DMXL1 siRNA sense sequences are as follows: 5′-GAAUGACAUUCAGUUGGCUCUUGUA-3′, 5′-CAGUAUAUCUUAGUCUGUUCAUUCA-3′, 5′-CCACUGGUGUGAACCAGUUAGUUUA-3′; DMXL2 siRNA sense sequences are: 5′-CCCAGUGUGCCUUGCACCUUAUUUA-3′, 5′-CAACUACCUUCGAACACAUCCUUUA-3′, 5′-CAGGACGGCAGUGUCCGCAUGUUUG-3′; WDR7 siRNA sense sequences are: 5′-CCCAGACGGCCAUAGUUCAGCUAUU-3′, 5′-GCAAGAGUUCAACACUGCAUCUGUU-3′, 5′-UAGGCCUCCUAAGUCUGCGAGAGAA-3′; ATP6V1A siRNA sense sequences are: 5′-GAGAGGCAACGAGAUGUCAGAAGUU-3′, 5′-CAACGCUGGGUAUUGUUCAGGUGUU-3′, 5′-CAGCGAGCUGGUUGGAGAAAUUAUU-3′; finally the negative control siRNA sense sequence is 5′-GGAGUCACGCGAUCGUGACGCGCCA-3′. In preliminary experiments the efficiency of knockdowns with these siRNAs was estimated at 72 h by quantitative real-time PCR, as described in the above section. For quantitative acidification assays, M-1 cells (ATCC® CRL-2038™, American Type Culture Collection (ATCC), Manassas, VA) were seeded in 24-well cell culture plates. The next day, they were transfected with a mixture of all three siRNAs per gene (40 nM each) or 120 nM of negative control siRNA using Lipofectamine 2000 transfection reagent (Invitrogen). Seventy-two hours after transfection, for each siRNA set, 2 wells (for duplicates) were left untreated (Control), two wells were pre-incubated with 100 nM bafilomycin A1 (Sigma-Aldrich, St. Louis, MO) for 1 h and two wells pre-incubated with 100 nM bafilomycin A1 for 1 h, which was then washed out for 2 h before being labeled with 1 μM LysoTracker for 1 h (total washout time is 3 hours). After labeling, cells in all wells were rinsed twice in PBS, lysed in 200 μl of RIPA lysis buffer (50 mM Tris-HCl, pH 7.4; 150 mM NaCl, 1% NP-40, 0.5% sodium deoxycholate, 0.1% SDS); the lysate was then transferred into a black 96-well plate (Corning, Corning, NY). Fluorescence released into the medium was read immediately using a Multimode Detector plate reader (model DTX 880; Beckman-Coulter, Fullerton, CA). The following settings were used: fluorescence intensity top method, 0.4-s integration time, 535-nm excitation filter, and 595-nm emission filter. In parallel, 20 μl of the same RIPA lysate were used to determine the total protein amount in each sample with the use of the Pierce™ BCA Protein Assay Kit (Thermo Fisher Scientific, Waltham, MA). Fluorescence values were then normalized to the total protein amount, and then to untreated (no bafilomycin) control values of negative control siRNA-treated M-1 cells, which were taken as 100%. The final reported values represent means of at least three independent experiments, measured in duplicate. For statistical analysis the recovery of acidification was first calculated as a ratio of each Baf-subtracted value at 3 hours after Baf washout to the corresponding Baf-subtracted untreated control values, presented as a percentage. Statistical significance was determined by one-way analysis of variance (ANOVA) with Bonferroni multiple comparison test using Prism 5 software (GraphPad Software, La Jolla, CA). P < 0.05 was considered significant. Graphs were plotted with Microsoft Excel for Mac 2011 software (Microsoft, Redmond, WA).

### Immunohistochemistry and epifluorescence microscopy

Fixation of mouse kidneys, preparation of cryostat sections, antigen retrieval, permeabilization and incubation with antibodies were performed as described elsewhere^34^,^47^,^48^. Affinity-purified rabbit polyclonal antibodies were used at the following dilutions: anti-DMXL1 (1:10), anti-DMXL2 (1:20), anti-NCOA7 (1:100), anti-OXR1 (1:50) and anti-WDR7 (1:50). The affinity-purified chicken polyclonal anti-ATP6V1A antibody was used at 1:800. Secondary Cy3-conjugated donkey anti-rabbit IgG (Jackson ImmunoResearch Laboratories, West Grove, PA) and Alexa Fluor® 594-conjugated goat anti-rabbit IgG (Life Technologies/Thermo Fisher Scientific) antibodies were used at 1:800, an FITC-conjugated donkey anti-chicken IgY antibody (Jackson ImmunoResearch) at 1:60 and an Alexa Fluor® 488-conjugated donkey anti-chicken IgY antibody (Jackson ImmunoResearch) at 1:200. Images were obtained using a Nikon 80i epifluorescence microscope (Nikon Instruments, Melville, NY) equipped with an Orca 100 CCD camera (Hamamatsu, Bridgewater, NJ).

## Additional Information

**How to cite this article**: Merkulova, M. *et al*. Mapping the H^+^(V)-ATPase interactome: identification of proteins involved in trafficking, folding, assembly and phosphorylation. *Sci. Rep*. **5**, 14827; doi: 10.1038/srep14827 (2015).

## Supplementary Material

Supplementary Information

Supplementary Table S1

Supplementary Table S2

Supplementary Table S3

Supplementary Table S4

Supplementary Table S5

## Figures and Tables

**Table 1:** List of proteins used to construct the V-ATPase interactome.

Official Symbol(MGI)	Official Name (MGI)	Proteins from V-ATPase interactome that associatewith each protein listed in column1
1. Transporters (19 proteins)		
*1.1. V-ATPase (18 proteins)*		
* *ATP6AP1	ATPase, H^^+^^ transporting, lysosomal accessory protein 1	all other subunits of V-ATPase^1^,^2^
* *ATP6AP2	ATPase, H^+^ transporting, lysosomal accessory protein 2	all other subunits of V-ATPase^1^,^2^
* *ATP6V0A1	ATPase, H^+^ transporting, lysosomal V0 subunit A1	all other subunits of V-ATPase^1^,^2^
* *ATP6V0A4	ATPase, H^+^ transporting, lysosomal V0 subunit A4	all other subunits of V-ATPase^1^,^2^
* *ATP6V0C	ATPase, H^+^ transporting, lysosomal V0 subunit C	all other subunits of V-ATPase^1^,^2^, SLC10A2^25^
* *ATP6V0D1	ATPase, H^+^ transporting, lysosomal V0 subunit D1	all other subunits of V-ATPase^1^,^2^
* *ATP6V0D2	ATPase, H^+^ transporting, lysosomal V0 subunit D2	all other subunits of V-ATPase^1^,^2^
* *ATP6V1A	ATPase, H^+^ transporting, lysosomal V1 subunit A	all other subunits of V-ATPase^1^,^2^
* *ATP6V1B1	ATPase, H^+^ transporting, lysosomal V1 subunit B1	all other subunits of V-ATPase^1^,^2^, ARHGEF7 (this study), CCT3 (this study), DMXL1 (this study), DMXL2 (this study), EZR (this study), NCOA7 (this study), OXR1 (this study), RPS6KA3 (this study), SLC10A2 (this study), SLC9A3R1^18^, SLC9A3R2 (this study), SNX27 (this study), TCP1 (this study), WDR7 (this study)
* *ATP6V1B2	ATPase, H^+^ transporting, lysosomal V1 subunit B2	all other subunits of V-ATPase^1^,^2^
* *ATP6V1C1	ATPase, H^+^ transporting, lysosomal V1 subunit C1	all other subunits of V-ATPase^1^,^2^, DMXL2^55^, WDR7^55^
* *ATP6V1C2	ATPase, H^+^ transporting, lysosomal V1 subunit C2	all other subunits of V-ATPase^1^,^2^
* *ATP6V1D	ATPase, H^+^ transporting, lysosomal V1 subunit D	all other subunits of V-ATPase^1^,^2^
* *ATP6V1E1	ATPase, H^+^ transporting, lysosomal V1 subunit E1	all other subunits of V-ATPase^1^,^2^, SLC9A3R1^18^, SLC9A3R2^18^
* *ATP6V1F	ATPase, H^+^ transporting, lysosomal V1 subunit F	all other subunits of V-ATPase^1^,^2^
* *ATP6V1G1	ATPase, H^+^ transporting, lysosomal V1 subunit G1	all other subunits of V-ATPase^1^,^2^
* *ATP6V1G3	ATPase, H^+^ transporting, lysosomal V1 subunit G3	all other subunits of V-ATPase^1^,^2^
* *ATP6V1H	ATPase, H^+^ transporting, lysosomal V1 subunit H	all other subunits of V-ATPase^1^,^2^
*1.2. Sodium-dependent bile acid transporter (1 protein)*		
* *SLC10A2	solute carrier family 10, member 2	ATP6V0C^25^, ATP6V1B1 (this study)
2. Chaperonin containing TCP1 complex (CCT) (9 proteins)		
* *CCT2	chaperonin containing Tcp1, subunit 2 (beta)	all other subunits of CCT^27^
* *CCT3	chaperonin containing Tcp1, subunit 3 (gamma)	all other subunits of CCT^27^, ATP6V1B1 (this study)
* *CCT4	chaperonin containing Tcp1, subunit 4 (delta)	all other subunits of CCT^27^
* *CCT5	chaperonin containing Tcp1, subunit 5 (epsilon)	all other subunits of CCT^27^
* *CCT6A	chaperonin containing Tcp1, subunit 6a (zeta)	all other subunits of CCT^27^
* *CCT6B	chaperonin containing Tcp1, subunit 6b (zeta)	all other subunits of CCT^27^
* *CCT7	chaperonin containing Tcp1, subunit 7 (eta)	all other subunits of CCT^27^
* *CCT8	chaperonin containing Tcp1, subunit 8 (theta)	all other subunits of CCT^27^
* *TCP1	t-complex protein 1	all other subunits of CCT^27^, ATP6V1B1 (this study)
3. Trafficking (6 proteins)		
* *ARHGEF7	Rho guanine nucleotide exchange factor (GEF7)	ATP6V1B1 (this study), SNX27^56^
* *EZR	ezrin	ATP6V1B1 (this study), SLC9A3R1^57^, SLC9A3R2^58^, SNX27^59^
* *RPS6KA3	ribosomal protein S6 kinase polypeptide 3	ATP6V1B1 (this study), SNX27^60^
* *SLC9A3R1	solute carrier family 9 (sodium/hydrogen exchanger), member 3 regulator 1	ATP6V1B1^18^, ATP6V1E1^18^, EZR^57^, SLC9A3R2^59^, SNX27^59^
* *SLC9A3R2	solute carrier family 9 (sodium/hydrogen exchanger), member 3 regulator 2	ATP6V1B1 (this study), ATP6V1E1^18^, EZR^58^, SLC9A3R1^59^, SNX27^59^
* *SNX27	sorting nexin family member 27	ARHGEF7^56^, ATP6V1B1 (this study), EZR^59^, RPS6KA3^60^, SLC9A3R1^59^, SLC9A3R2^59^
4. V-ATPase-specific accessory proteins (5 proteins)		
* *DMXL1	Dmx-like 1	ATP6V1B1 (this study)
* *DMXL2	Dmx-like 2	ATP6V1B1 (this study), ATP6V1C1^55^, WDR7^39^,^55^
* *NCOA7	nuclear receptor coactivator 7	ATP6V1B1 (this study)
* *OXR1	oxidation resistance 1	ATP6V1B1 (this study)
* *WDR7	WD repeat domain 7	ATP6V1B1 (this study), ATP6V1C1^55^, DMXL2^39^,^55^

Proteins are assigned to one of four major functional groups. Within each group proteins are shown in alphabetical order.

## References

[b1] Brown D., Paunescu T. G., Breton S. & Marshansky V. Regulation of the V-ATPase in kidney epithelial cells: dual role in acid-base homeostasis and vesicle trafficking. J Exp Biol 212, 1762–1772 (2009). 19448085 10.1242/jeb.028803 PMC2683016

[b2] Forgac M. Vacuolar ATPases: rotary proton pumps in physiology and pathophysiology. Nat Rev Mol Cell Biol 8, 917–929 (2007). 17912264 10.1038/nrm2272

[b3] Wagner C. A. . Renal vacuolar H^+^-ATPase. Physiol Rev 84, 1263–1314 (2004). 15383652 10.1152/physrev.00045.2003

[b4] Breton S. & Brown D. Regulation of luminal acidification by the V-ATPase. Physiology (Bethesda) 28, 318–329 (2013). 10.1152/physiol.00007.2013 PMC3768094 23997191

[b5] Marshansky V., Rubinstein J. L. & Gruber G. Eukaryotic V-ATPase: novel structural findings and functional insights. Biochim Biophys Acta 1837, 857–879 (2014). 24508215 10.1016/j.bbabio.2014.01.018

[b6] Hurtado-Lorenzo A. . V-ATPase interacts with ARNO and Arf6 in early endosomes and regulates the protein degradative pathway. Nat Cell Biol 8, 124–136 (2006). 16415858 10.1038/ncb1348

[b7] Merkulova M., Bakulina A., Thaker Y. R., Gruber G. & Marshansky V. Specific motifs of the V-ATPase a2-subunit isoform interact with catalytic and regulatory domains of ARNO. Biochim Biophys Acta 1797, 1398–1409 (2010). 20153292 10.1016/j.bbabio.2010.02.009

[b8] Hosokawa H. . The N termini of a-subunit isoforms are involved in signaling between vacuolar H^+^-ATPase (V-ATPase) and cytohesin-2. J Biol Chem 288, 5896–5913 (2013). 23288846 10.1074/jbc.M112.409169 PMC3581410

[b9] Schwartz J. H. . Role of SNAREs and H^+^-ATPase in the targeting of proton pump-coated vesicles to collecting duct cell apical membrane. Kidney Int 72, 1310–1315 (2007). 17805241 10.1038/sj.ki.5002500

[b10] Sun-Wada G. H. & Wada Y. Vacuolar-type proton pump ATPases: roles of subunit isoforms in physiology and pathology. Histol Histopathol 25, 1611–1620 (2010). 20886440 10.14670/HH-25.1611

[b11] Sun-Wada G. . Acidic endomembrane organelles are required for mouse postimplantation development. Dev Biol 228, 315–325 (2000). 11112332 10.1006/dbio.2000.9963

[b12] Miranda K. C., Karet F. E. & Brown D. An extended nomenclature for mammalian V-ATPase subunit genes and splice variants. PLoS One 5, e9531 (2010). 20224822 10.1371/journal.pone.0009531 PMC2835735

[b13] Kartner N. & Manolson M. F. V-ATPase subunit interactions: the long road to therapeutic targeting. Curr Protein Pept Sci 13, 164–179 (2012). 22044155 10.2174/138920312800493179

[b14] Korvatska O. . Altered splicing of ATP6AP2 causes X-linked parkinsonism with spasticity (XPDS). Hum Mol Genet 22, 3259–3268 (2013). 23595882 10.1093/hmg/ddt180 PMC3723311

[b15] Sun-Wada G. H. . The a3 isoform of V-ATPase regulates insulin secretion from pancreatic beta-cells. J Cell Sci 119, 4531–4540 (2006). 17046993 10.1242/jcs.03234

[b16] Sennoune S. R. & Martinez-Zaguilan R. Vacuolar H(^+^)-ATPase signaling pathway in cancer. Curr Protein Pept Sci 13, 152–163 (2012). 22044157 10.2174/138920312800493197

[b17] Kane P. M. Targeting reversible disassembly as a mechanism of controlling V-ATPase activity. Curr Protein Pept Sci 13, 117–123 (2012). 22044153 10.2174/138920312800493142 PMC3536023

[b18] Breton S. . The B1 subunit of the H^+^ ATPase is a PDZ domain-binding protein. Colocalization with NHE-RF in renal B-intercalated cells. J Biol Chem 275, 18219–18224 (2000). 10748165 10.1074/jbc.M909857199

[b19] Finberg K. E. . The B1-subunit of the H(^+^) ATPase is required for maximal urinary acidification. Proc Natl Acad Sci USA 102, 13616–13621 (2005). 16174750 10.1073/pnas.0506769102 PMC1224669

[b20] Pietrement C. . Role of NHERF1, cystic fibrosis transmembrane conductance regulator, and cAMP in the regulation of aquaporin 9. J Biol Chem 283, 2986–2996 (2008). 18055461 10.1074/jbc.M704678200

[b21] Mellacheruvu D. . The CRAPome: a contaminant repository for affinity purification-mass spectrometry data. Nat Methods 10, 730–736 (2013). 23921808 10.1038/nmeth.2557 PMC3773500

[b22] Saito R. . A travel guide to Cytoscape plugins. Nat Methods 9, 1069–1076 (2012). 23132118 10.1038/nmeth.2212 PMC3649846

[b23] Sun-Wada G. H. . Mouse proton pump ATPase C subunit isoforms (C2-a and C2-b) specifically expressed in kidney and lung. J Biol Chem 278, 44843–44851 (2003). 12947086 10.1074/jbc.M307197200

[b24] Lee S. H. . v-ATPase V0 subunit d2-deficient mice exhibit impaired osteoclast fusion and increased bone formation. Nat Med 12, 1403–1409 (2006). 17128270 10.1038/nm1514

[b25] Sun A. Q., Balasubramaniyan N., Liu C. J., Shahid M. & Suchy F. J. Association of the 16-kDa subunit c of vacuolar proton pump with the ileal Na^+^ -dependent bile acid transporter: protein-protein interaction and intracellular trafficking. J Biol Chem 279, 16295–16300 (2004). 14752118 10.1074/jbc.M312838200

[b26] Chambrey R. . Renal intercalated cells are rather energized by a proton than a sodium pump. Proc Natl Acad Sci USA 110, 7928–7933 (2013). 23610411 10.1073/pnas.1221496110 PMC3651478

[b27] Hartl F. U. & Hayer-Hartl M. Molecular chaperones in the cytosol: from nascent chain to folded protein. *Science* 295, 1852–1858 (2002). 10.1126/science.1068408 11884745

[b28] Ramachandran N. . VMA21 deficiency prevents vacuolar ATPase assembly and causes autophagic vacuolar myopathy. Acta Neuropathol 125, 439–457 (2013). 23315026 10.1007/s00401-012-1073-6

[b29] Chen S. H. . Vacuolar H^+^-ATPase binding to microfilaments: regulation in response to phosphatidylinositol 3-kinase activity and detailed characterization of the actin-binding site in subunit B. J Biol Chem 279, 7988–7998 (2004). 14662773 10.1074/jbc.M305351200

[b30] Beaulieu V. . Modulation of the actin cytoskeleton via gelsolin regulates vacuolar H^+^-ATPase recycling. J Biol Chem 280, 8452–8463 (2005). 15591047 10.1074/jbc.M412750200

[b31] Holliday L. S. . The amino-terminal domain of the B subunit of vacuolar H^+^-ATPase contains a filamentous actin binding site. J Biol Chem 275, 32331–32337 (2000). 10915794 10.1074/jbc.M004795200

[b32] Vitavska O., Wieczorek H. & Merzendorfer H. A novel role for subunit C in mediating binding of the H^+^-V-ATPase to the actin cytoskeleton. J Biol Chem 278, 18499–18505 (2003). 12606563 10.1074/jbc.M212844200

[b33] Steinberg F. . A global analysis of SNX27-retromer assembly and cargo specificity reveals a function in glucose and metal ion transport. Nat Cell Biol 15, 461–471 (2013). 23563491 10.1038/ncb2721 PMC4052425

[b34] Paunescu T. G. . cAMP stimulates apical V-ATPase accumulation, microvillar elongation, and proton extrusion in kidney collecting duct A-intercalated cells. Am J Physiol Renal Physiol 298, F643–654 (2010). 20053793 10.1152/ajprenal.00584.2009 PMC2838605

[b35] Brown D., Bouley R., Paunescu T. G., Breton S. & Lu H. A. New insights into the dynamic regulation of water and acid-base balance by renal epithelial cells. Am J Physiol Cell Physiol 302, C1421–1433 (2012). 22460710 10.1152/ajpcell.00085.2012 PMC3362000

[b36] Alzamora R. . AMP-activated protein kinase regulates the vacuolar H^+^ -ATPase via direct phosphorylation of the A subunit (ATP6V1A) in the kidney. Am J Physiol Renal Physiol 305, F943–956 (2013). 23863464 10.1152/ajprenal.00303.2013 PMC3798744

[b37] Miller R. L. . V-ATPase B1-subunit promoter drives expression of EGFP in intercalated cells of kidney, clear cells of epididymis and airway cells of lung in transgenic mice. Am J Physiol Cell Physiol 288, C1134–1144 (2005). 15634743 10.1152/ajpcell.00084.2004

[b38] Vedovelli L. . Altered V-ATPase expression in renal intercalated cells isolated from B1 subunit-deficient mice by fluorescence-activated cell sorting. Am J Physiol Renal Physiol 304, F522–532 (2013). 23269648 10.1152/ajprenal.00394.2012 PMC3602708

[b39] Kawabe H. . A novel rabconnectin-3-binding protein that directly binds a GDP/GTP exchange protein for Rab3A small G protein implicated in Ca(2^+^)-dependent exocytosis of neurotransmitter. Genes Cells 8, 537–546 (2003). 12786944 10.1046/j.1365-2443.2003.00655.x

[b40] Sethi N., Yan Y., Quek D., Schupbach T. & Kang Y. Rabconnectin-3 is a functional regulator of mammalian Notch signaling. J Biol Chem 285, 34757–34764 (2010). 20810660 10.1074/jbc.M110.158634 PMC2966091

[b41] Behrends C., Sowa M. E., Gygi S. P. & Harper J. W. Network organization of the human autophagy system. Nature 466, 68–76 (2010). 20562859 10.1038/nature09204 PMC2901998

[b42] Kristensen A. R., Gsponer J. & Foster L. J. A high-throughput approach for measuring temporal changes in the interactome. Nat Methods 9, 907–909 (2012). 22863883 10.1038/nmeth.2131 PMC3954081

[b43] Volkert M. R., Elliott N. A. & Housman D. E. Functional genomics reveals a family of eukaryotic oxidation protection genes. Proc Natl Acad Sci USA 97, 14530–14535 (2000). 11114193 10.1073/pnas.260495897 PMC18953

[b44] Elliott N. A. & Volkert M. R. Stress induction and mitochondrial localization of Oxr1 proteins in yeast and humans. Mol Cell Biol 24, 3180–3187 (2004). 15060142 10.1128/MCB.24.8.3180-3187.2004 PMC381681

[b45] Hutagalung A. H. & Novick P. J. Role of Rab GTPases in membrane traffic and cell physiology. Physiol Rev 91, 119–149 (2011). 21248164 10.1152/physrev.00059.2009 PMC3710122

[b46] Einhorn Z., Trapani J. G., Liu Q. & Nicolson T. Rabconnectin3alpha promotes stable activity of the H^+^ pump on synaptic vesicles in hair cells. J Neurosci 32, 11144–11156 (2012). 22875945 10.1523/JNEUROSCI.1705-12.2012 PMC3428958

[b47] Paunescu T. G. . Expression of the 56-kDa B2 subunit isoform of the vacuolar H(^+^)-ATPase in proton-secreting cells of the kidney and epididymis. Am J Physiol Cell Physiol 287, C149–162 (2004). 15013950 10.1152/ajpcell.00464.2003

[b48] Paunescu T. G. . Association of soluble adenylyl cyclase with the V-ATPase in renal epithelial cells. Am J Physiol Renal Physiol 294, F130–138 (2008). 17959750 10.1152/ajprenal.00406.2007

[b49] Shevchenko A., Wilm M., Vorm O. & Mann M. Mass spectrometric sequencing of proteins silver-stained polyacrylamide gels. Anal Chem 68, 850–858 (1996). 8779443 10.1021/ac950914h

[b50] Peng J. & Gygi S. P. Proteomics: the move to mixtures. J Mass Spectrom 36, 1083–1091 (2001). 11747101 10.1002/jms.229

[b51] Eng J. K., McCormack A. L. & Yates J. R. An approach to correlate tandem mass spectral data of peptides with amino acid sequences in a protein database. J Am Soc Mass Spectrom 5, 976–989 (1994). 24226387 10.1016/1044-0305(94)80016-2

[b52] Choi H. . SAINT: probabilistic scoring of affinity purification-mass spectrometry data. Nat Methods 8, 70–73 (2011). 21131968 10.1038/nmeth.1541 PMC3064265

[b53] Razick S., Magklaras G. & Donaldson I. M. iRefIndex: a consolidated protein interaction database with provenance. BMC Bioinformatics 9, 405 (2008). 18823568 10.1186/1471-2105-9-405 PMC2573892

[b54] Da Silva N. . Proteomic analysis of V-ATPase-rich cells harvested from the kidney and epididymis by fluorescence-activated cell sorting. Am J Physiol Cell Physiol 298, C1326–1342 (2010). 20181927 10.1152/ajpcell.00552.2009 PMC2889637

[b55] Li K. W. . Identifying true protein complex constituents in interaction proteomics: the example of the DMXL2 protein complex. Proteomics 12, 2428–2432 (2012). 22707207 10.1002/pmic.201100675

[b56] Valdes J. L. . Sorting nexin 27 protein regulates trafficking of a p21-activated kinase (PAK) interacting exchange factor (beta-Pix)-G protein-coupled receptor kinase interacting protein (GIT) complex via a PDZ domain interaction. J Biol Chem 286, 39403–39416 (2011). 21926430 10.1074/jbc.M111.260802 PMC3234764

[b57] Reczek D., Berryman M. & Bretscher A. Identification of EBP50: A PDZ-containing phosphoprotein that associates with members of the ezrin-radixin-moesin family. J Cell Biol 139, 169–179 (1997). 9314537 10.1083/jcb.139.1.169 PMC2139813

[b58] Yun C. H., Lamprecht G., Forster D. V. & Sidor A. NHE3 kinase A regulatory protein E3KARP binds the epithelial brush border Na^+^/H^+^ exchanger NHE3 and the cytoskeletal protein ezrin. J Biol Chem 273, 25856–25863 (1998). 9748260 10.1074/jbc.273.40.25856

[b59] Arnaud C. . MCC, a new interacting protein for Scrib, is required for cell migration in epithelial cells. FEBS Lett 583, 2326–2332 (2009). 19555689 10.1016/j.febslet.2009.06.034

[b60] Cai L., Loo L. S., Atlashkin V., Hanson B. J. & Hong W. Deficiency of sorting nexin 27 (SNX27) leads to growth retardation and elevated levels of N-methyl-D-aspartate receptor 2C (NR2C). Mol Cell Biol 31, 1734–1747 (2011). 21300787 10.1128/MCB.01044-10 PMC3126336

