# Supplementary material for: Mapping the H+ (V)-ATPase interactome: identification of proteins involved in trafficking, folding, assembly and phosphorylation
Source: Sci Rep. 2015 Oct 7;5:14827. doi: 10.1038/srep14827 (PMC4595830; doi:10.1038/srep14827)
Supplement: Supplementary Information [file srep14827-s1.pdf]

Supplementary Information for:

Mapping the H^+(V)-ATPase interactome: identification of proteins involved in trafficking, folding, assembly and phosphorylation.

Maria Merkulova, Teodor G. Păunescu, Anie Azroyan, Vladimir Marshansky, Sylvie Breton and Dennis Brown

MGH Center for Systems Biology, Program in Membrane Biology & Division of Nephrology, Richard B. Simches Research Center, Massachusetts General Hospital and Department of Medicine, Harvard Medical School, Boston, MA 02114, USA

Correspondence should be addressed to:

D. Brown, Ph. D.

Program in Membrane Biology and Division of Nephrology

Simches Research Center

185 Cambridge Street,

Boston, MA 02114

e-mail: brown.dennis@mgh.harvard.edu

Tel: (617) 726-5665

Supplementary Figure S1. Visualization of the V-ATPase interactome, based on previously reported interactions of all known subunits of V-ATPase. QIAGEN's Ingenuity® Pathway Analysis (IPA®) was used first to connect 25 subunits of the V-ATPase with each other, and then their first neighbors were found with the "grow" function of IPA®. In total, 176 proteins were identified as previously reported to interact with one or more subunits of the V-ATPase. These 176 V-ATPase-interacting proteins and 25 V-ATPase subunits are shown as 201 nodes. Among them, 18 proteins (including 13 V-ATPase subunits) were also found in this study with SAINT≥0.67 (threshold for specific interactions) for ATP6V1B1_ΔCterminus interactome or SAINT≥0.99 (threshold for specific interactions) for ATP6V1B1_Cterminus interactome, and are circled in red. 49 proteins (including 5 V-ATPase subunits) were also detected in this study but had lower scores: SAINT<0.67 for ATP6V1B1_ΔCterminus interactome and SAINT<0.99 for ATP6V1B1_Cterminus interactome (circled in green). Proteins that are not detected in this study at all or were present in negative controls only are not circled. See also Supplementary Table S5 for further details.

Supplementary Figure S1

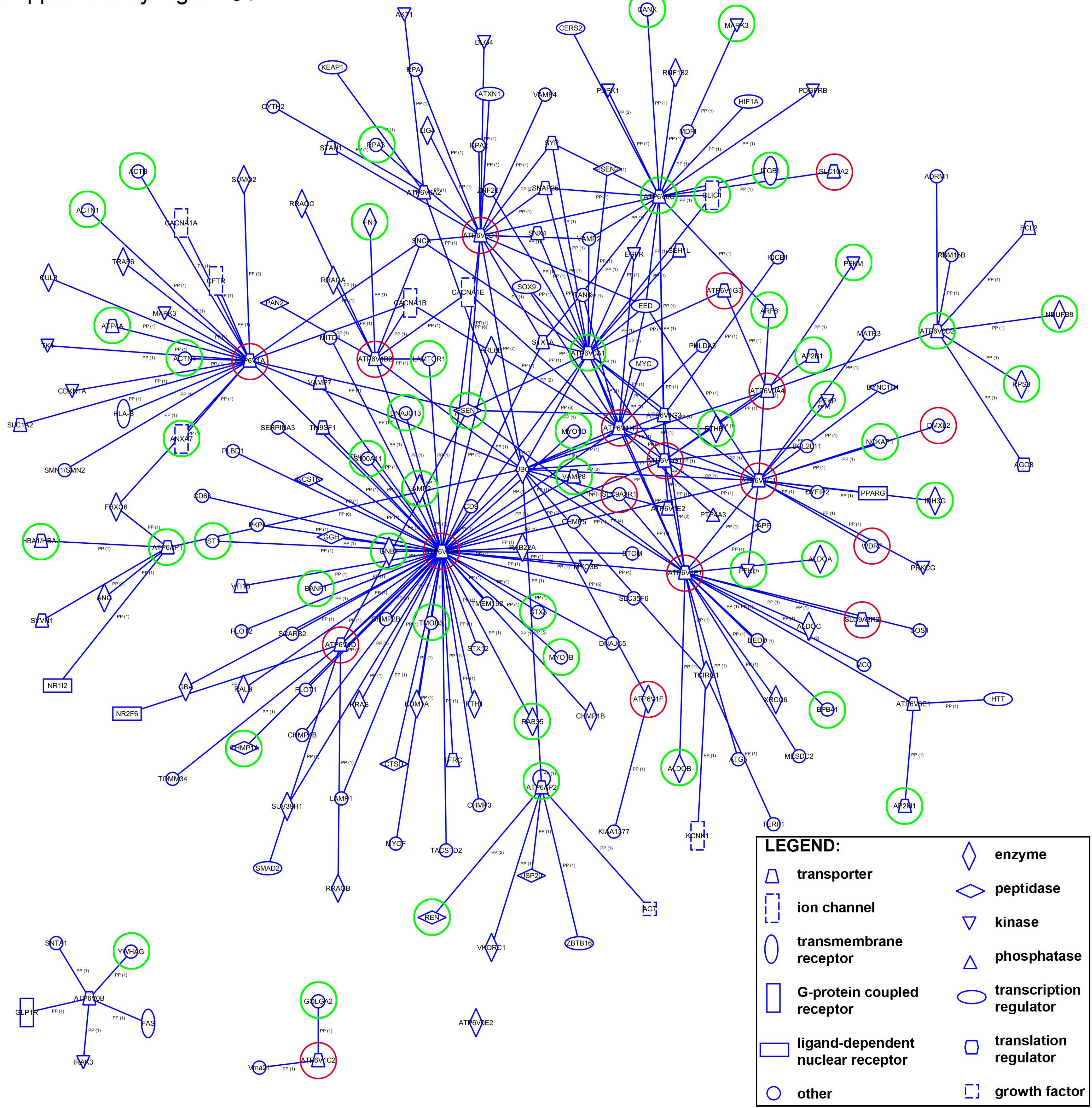

SUPPLEMENTARY TABLE LEGENDS.

Note: All Supplementary tables are Microsoft Excel files uploaded as separate Supplementary Datasets.

Supplementary Table S1. Summary of all LC-MS/MS datasets, used in this study to construct the V-ATPase interactome. In total, 11 datasets (IP1, IP2, IP3, IP4 replicates; IP1beads, IP4beads, IP5beads replicates; IP2peptide, IP3peptide replicates and IP6KO, IP7KO replicates) were used for scoring of protein-protein interactions and construction of the interactome. Reproducibility of experimental and negative control replicates is shown at the bottom of the table.

Supplementary Table S2. The list of all proteins and peptides identified by SEQUEST (ThermoFisher, San Jose, CA). Each dataset (IP1, IP2, IP3, IP4, IP1beads, IP4beads, IP5beads, IP2peptide, IP3peptide, IP6KO and IP7KO) is shown on a separate sheet (a total of 11 datasets). The protein lists are shown on the left of each sheet, including UNIPROT or IPI IDs, average Xcorr, unique peptide counts and total spectrum counts, and are sorted in descending order of their total spectrum counts. The peptide lists are shown on the right of each sheet, include UNIPROT or IPI IDs, peptide sequences, redundancy, charge, Xcorr, deltaCorr and ions, and are sorted in alphabetical order of UNIPROT or IPI IDs.

Supplementary Table S3. ATP6V1B1_ΔCterminus interactome scoring with the CRAPome computational tools (http://www.crapome.org/). IP1, IP2, IP3 and IP4 experiments served as baits and scored with all other 7 experiments (IP1beads, IP4beads, IP5beads, IP2peptide, IP3peptide, IP6KO and IP7KO) as negative controls. Official protein symbols and names are shown in accordance with the nomenclature from the Mouse Genome Informatics (MGI) database. Total spectrum counts are shown for each protein across all the experiments along with FC-A, FC-B and SAINT scores, calculated by the CRAPome. Previously known interactions deposited in iRefIndex database are denoted in the last

column. Proteins are sorted in the descending order of their SAINT scores and then FC-B scores.

Supplementary Table S4. ATP6V1B1_Cterminus interactome scoring with the CRAPome computational tools. IP2peptide and IP3peptide experiments served as baits and scored with IP2 and IP3 as negative controls. Official protein symbols and names are shown in accordance with the nomenclature from the Mouse Genome Informatics (MGI) database. Total spectrum counts are shown for each protein across all four experiments along with FC-A, FC-B and SAINT scores, calculated by the CRAPome. Previously known interactions deposited in iRefIndex database are denoted in the last column. Proteins are sorted in descending order of their SAINT scores and then FC-B scores.

Supplementary Table S5. Comparison of previously reported V-ATPase protein-protein interactions with the results of this study. Among 201 proteins (25 subunits of V-ATPase and 176 previously reported V-ATPase interacting proteins, found by IPA®) 130 (including 7 subunits of V-ATPase) were not detected in this study at all, they are shown at the bottom of the table in alphabetical order. 71 proteins were detected in this study and are shown at the top of the table in descending order of their SAINT scores, calculated by the CRAPome computational tools. In this study, proteins with SAINT≥0.67 for ATP6V1B1_ΔCterminus interactome or SAINT≥0.99 for ATP6V1B1_Cterminus interactome were considered specific interactors (highlighted in light-green). Note, that 4 detected proteins were present in negative controls only (indicated by asterisk). Note also, that some official MGI symbols are different from IPA® symbols (indicated by bold font style).
